# CRISPR/Cas9-mediated reversibly immortalized mouse bone marrow stromal stem cells (BMSCs) retain multipotent features of mesenchymal stem cells (MSCs)

**DOI:** 10.18632/oncotarget.22915

**Published:** 2017-12-05

**Authors:** Xue Hu, Li Li, Xinyi Yu, Ruyi Zhang, Shujuan Yan, Zongyue Zeng, Yi Shu, Chen Zhao, Xingye Wu, Jiayan Lei, Yasha Li, Wenwen Zhang, Chao Yang, Ke Wu, Ying Wu, Liping An, Shifeng Huang, Xiaojuan Ji, Cheng Gong, Chengfu Yuan, Linghuan Zhang, Wei Liu, Bo Huang, Yixiao Feng, Bo Zhang, Rex C. Haydon, Hue H. Luu, Russell R. Reid, Michael J. Lee, Jennifer Moriatis Wolf, Zebo Yu, Tong-Chuan He

**Affiliations:** ^1^ Departments of Blood Transfusion, Nephrology, Orthopaedic Surgery, and General Surgery, The First Affiliated Hospital of Chongqing Medical University, Chongqing 400016, China; ^2^ Molecular Oncology Laboratory, Department of Orthopaedic Surgery and Rehabilitation Medicine, The University of Chicago Medical Center, Chicago, IL 60637, USA; ^3^ Ministry of Education Key Laboratory of Diagnostic Medicine, and School of Laboratory Medicine, Chongqing Medical University, Chongqing 400016, China; ^4^ Department of Biomedical Engineering, School of Biomedical Engineering, Chongqing University, Chongqing 400044, China; ^5^ The Children’s Hospital, Chongqing Medical University, Chongqing 400014, China; ^6^ Department of Laboratory Medicine and Clinical Diagnostics, The Affiliated Yantai Hospital, Binzhou Medical University, Yantai 264100, China; ^7^ Department of Immunology and Microbiology, Beijing University of Chinese Medicine, Beijing 100029, China; ^8^ Key Laboratory of Orthopaedic Surgery of Gansu Province and The Department of Orthopaedic Surgery, The Second Hospital of Lanzhou University, Lanzhou 730030, China; ^9^ Department of Surgery, The Affiliated Zhongnan Hospital of Wuhan University, Wuhan 430071, China; ^10^ Department of Biochemistry and Molecular Biology, China Three Gorges University School of Medicine, Yichang 443002, China; ^11^ Department of Surgery, Section of Plastic Surgery, The University of Chicago Medical Center, Chicago, IL 60637, USA

**Keywords:** mesenchymal stem cells (MSCs), bone marrow stromal stem cells (BMSCs), CRISPR/Cas9 genome-editing, BMP9, osteogenic differentiation

## Abstract

Mesenchymal stem cells (MSCs) are multipotent non-hematopoietic progenitor cells that can undergo self-renewal and differentiate into multi-lineages. Bone marrow stromal stem cells (BMSCs) represent one of the most commonly-used MSCs. In order to overcome the technical challenge of maintaining primary BMSCs in long-term culture, here we seek to establish reversibly immortalized mouse BMSCs (imBMSCs). By exploiting CRISPR/Cas9-based homology-directed-repair (HDR) mechanism, we target SV40T to mouse *Rosa26* locus and efficiently immortalize mouse BMSCs (i.e., imBMSCs). We also immortalize BMSCs with retroviral vector SSR #41 and establish imBMSC41 as a control line. Both imBMSCs and imBMSC41 exhibit long-term proliferative capability although imBMSC41 cells have a higher proliferation rate. SV40T mRNA expression is 130% higher in imBMSC41 than that in imBMSCs. However, FLP expression leads to 86% reduction of SV40T expression in imBMSCs, compared with 63% in imBMSC41 cells. Quantitative genomic PCR analysis indicates that the average copy number of SV40T and hygromycin is 1.05 for imBMSCs and 2.07 for imBMSC41, respectively. Moreover, FLP expression removes 92% of SV40T in imBMSCs at the genome DNA level, compared with 58% of that in imBMSC41 cells, indicating CRISPR/Cas9 HDR-mediated immortalization of BMSCs can be more effectively reversed than that of retrovirus-mediated random integrations. Nonetheless, both imBMSCs and imBMSC41 lines express MSC markers and are highly responsive to BMP9-induced osteogenic, chondrogenic and adipogenic differentiation *in vitro* and *in vivo*. Thus, the engineered imBMSCs can be used as a promising alternative source of primary MSCs for basic and translational research in the fields of MSC biology and regenerative medicine.

## INTRODUCTION

Considered as multipotent progenitors, mesen-chymal stem cells (MSCs) are a heterogeneous population of non-hematopoietic progenitor cells that can undergo self-renewal and differentiate into multi-lineages [1-5]. MSCs were originally described by Friedentstein and colleagues near 50 years ago as adherent cells with a fibroblast-like appearance capable of differentiating into osteocytes, chondrocytes, adipocytes, tenocytes and myocytes upon appropriate stimulations [6, 7]. For instance, osteogenic differentiation of MSCs is a cascade that recapitulates most, if not all, of the molecular events occurring during embryonic skeletal development [8]. While many signaling pathways play important roles in regulating osteogenic differentiation [4, 9-17], bone morphogenetic proteins (BMPs) are considered as a group of the most potent osteoinductive factors [4, 18-20]. We previously demonstrated that BMP9 (also known as growth differentiation factor 2, or GDF2) is one of the most potent BMPs among the 14 types of BMPs in inducing osteogenic differentiation of MSCs [21-24].

MSCs have attracted significant attention for their potential role in elucidating differentiation pathways, promoting tissue engineering and functioning as immunomodulators in autoimmune diseases [3, 4]. While MSCs can be isolated from various tissues [2, 3, 25], MSCs isolated from the bone marrow, a subset of bone marrow stromal cells, are the most characterized [1, 5]. Nonetheless, clinical translation of MSCs is hampered by many issues. One of the major technical challenges is to obtain adequate number of therapeutic cells, which requires expansion of MSCs while maintaining their stem cell phenotype *in vitro*. Even though BMSCs are one of the most commonly-used MSCs, their isolation and maintenance is time-consuming and labor-intensive. Thus, immortalized bone marrow stromal stem cells (BMSCs) can be a promising alternative cell source of primary MSCs for basic and pre-clinical studies.

One commonly-used strategy is to express an immortalizing oncogene in primary cells, which is exemplified by the use of simian virus SV40 large T antigen (SV40T) [26]. SV40T is a multifunctional regulatory protein which binds and inactivates p53 and Rb, at least in part leading to unlimited life span [26, 27]. Unlike most other oncogene products and owing to its multiple effects on the cell cycle, SV40T alone can immortalize cells [27]. It is important to have an effective method of delivering the immortalizing genes into the target cells. The common approaches include the use of transposons or retroviral (including lentiviral) vectors to achieve efficient and stable DNA integration. We previously used the retroviral vector and *piggyBac* transposon-mediated expression of SV40T and immortalized several sources of progenitor cells [28-39]. However, retroviral or transposon-mediated random integration of immortalizing genes into the host genome may possess detrimental effects. Thus, safer strategies of delivering immortalizing genes should be used.

The recent discovery of CRISPR/Cas9 genome-editing system provides us an unprecedented opportunity to target and modify genomic sequences with high levels of efficacy and specificity [40-44]. CRISPR/Cas9 system induces DNA double-strand breaks at specific sites of genomic DNA, which should allow safer and targeted gene delivery of the immortalizing genes. Numerous studies have identified “safe harbor” loci in human and mouse genomes, which can be specifically targeted without causing significant detrimental effects on host genes while maintaining a high level of gene expression. Mouse *Rosa26* locus is such a “safe harbor” locus for targeted integration because this site is not susceptible to gene silencing effects and provides improved targeting efficiency and ubiquitous transgene expression without alteration of the cell viability or phenotype [45, 46]. Furthermore, it is conceivable that such site-specific targeted integration of immortalization should allow more efficient removal of the immortalizing genes than that of random integrations.

In order to overcome the technical challenge of maintaining primary BMSCs in long-term culture, here we established and characterized the reversibly immortalized mouse BMSCs (imBMSCs) through the CRISPR/Cas9-mediated homology-directed-repair (HDR) mechanism. We demonstrated that mouse BMSCs were effectively immortalized by targeting SV40T into the *Rosa26* locus through CRISPR/Cas9 HDR and the resultant imBMSCs retained MSC-like features both *in vitro* and *in vivo*. Furthermore, the CRISPR/Cas9 HDR-immortalized BMSCs can be reversed more effectively by the FLP recombinase than that of the BMSCs immortalized with retroviral vector-based random integrations. Therefore, the engineered imBMSCs should be a valuable resource for basic and translational research in the fields of MSC biology and regenerative medicine.

## RESULTS

### Mouse bone marrow stromal stem cells (mBMSCs) can be immortalized by SV40 T antigen through CRISPR/Cas9-mediated targeted integration at the *Rosa26* locus

Bone marrow stromal stem cells (BMSCs) are a valuable cell type for a broad range of studies [2, 3]. While readily available, primary BMSCs are not easy to culture and grow to large quantities. Thus, there is a need to establish reversibly and/or conditionally immortalized BMSCs. While SV40 T antigen (SV40T) has been widely used to immortalize primary mammalian cells, this immortalizing gene is usually delivered by retroviral vectors or *piggyBac* transposon system [28, 29, 39, 47, 48], which often randomly integrate into host genome. Here, we sought to take advantage of the high genome-editing specificity feature delivered by CRISPR/Cas9 system and to target the SV40T into a safe harboring site at *Rosa26* locus [49].

**Figure 1:**
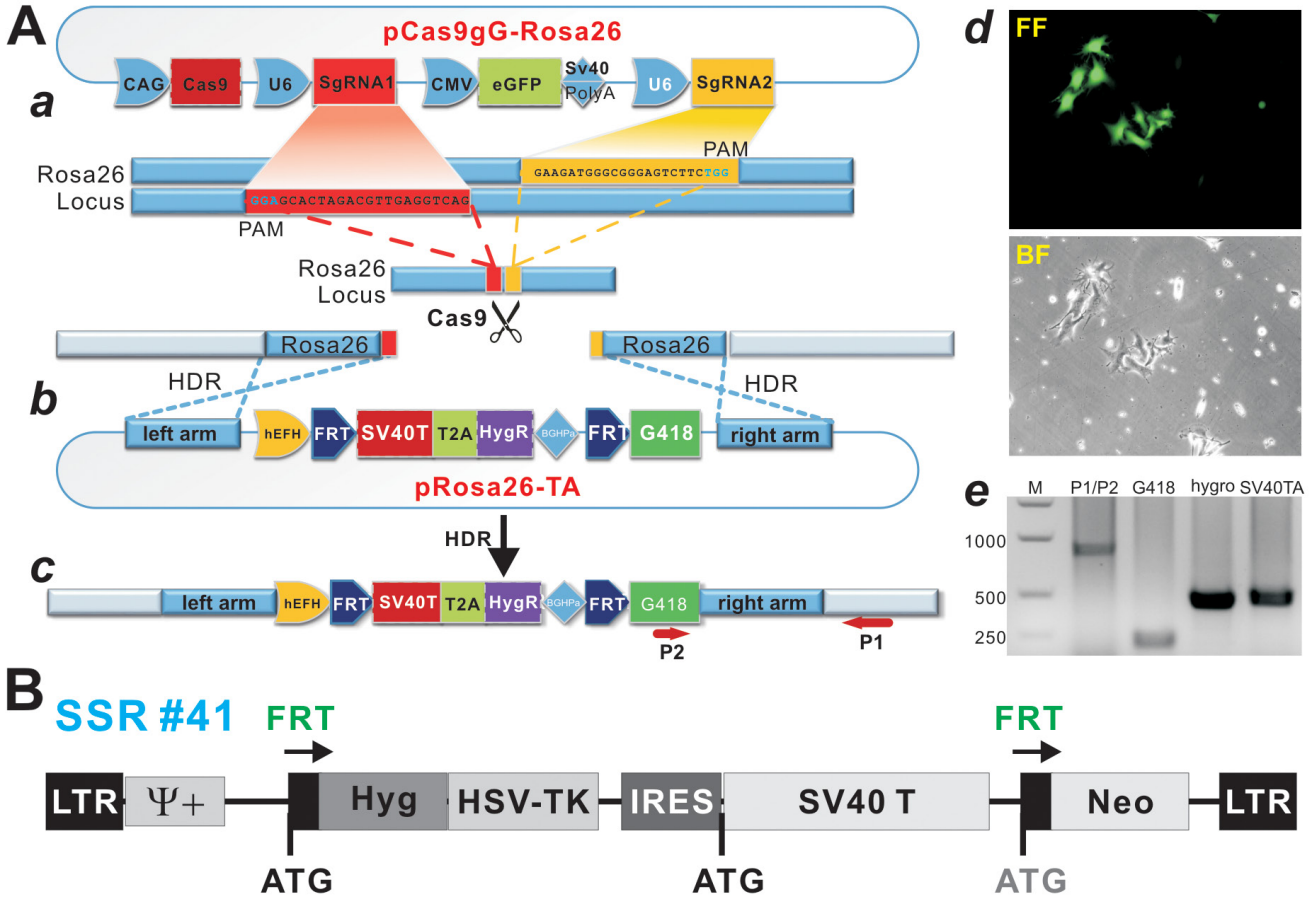
A CRISPR/Cas9-based SV40 T-antigen immortalization strategy by targeting *Rosa26* locus **(A)** Strategy of knocking-in SV40T and hygromycin resistance gene (HygR) into the *Rosa26* locus using the CRISPR/Cas9 homology-directed-repair (HDR) technique. ***(a)*** Schematic representation of the Cas9 expression vector pCas9gG-Rosa26, which expresses Cas9 and a pair of sgRNAs targeting mouse *Rosa26* locus, sgRNA1 (red) and sgRNA2 (yellow), each driven by a U6 promoter. The protospacer-adjacent motif (PAM) sequence (NGG) is in green. This vector also co-expresses eGFP for monitoring transfection efficiency. The sgRNA pairs will guide the Cas9 nuclease to the target sites and cleave genomic DNA. ***(b)*** Schematic of the donor plasmid pRosa26-TA, which consists of the hygromycin (HygR) and SV40 T antigen (SV40T) T2A fusion expression cassette flanked by the FRT sites and mouse *Rosa26* homology arms. ***(c)*** After co-transfection of pRosa26-TA with pCas9gG-Rosa26 into target cells, the guide RNA pairs and Cas9 introduce double-strand breaks in the targeting vector and the targeted locus. HDR leads to the insertion of the cassette into the genome. Arrows (P1 & P2) indicate the directions and locations of PCR primers for detecting successful targeting SV40T-HygR transgenes into *Rosa26* locus. ***(d)*** The proof-of-principle test of HDR in a mouse melanoma line. After co-transfected with pRosa26-TA and pCas9gG-Rosa26 vectors, the infected B16F10 cells were selected in 0.4mg/ml hygromycin B. At 5 days post selection, surviving clones were observed under fluorescence microscopy (top) and bright field (bottom). ***(e)*** PCR confirmation of CRISPR/Cas9-mediated HDR targeting of *Rosa26* locus. Genomic DNA was isolated from the stable cell pool and subjected to PCR analysis using primers to amplify the integration site (P1/P2) and transgenes (G418, hygro and SV40T). M, DNA ladder. **(B)** A retroviral vector expressing SV40T flanked with FRT sites. The previously reported retroviral SSR #41 vector system was used as a control [47].

To accomplish the efficient expression of Cas9 and *Rosa26* locus-targeting sgRNAs in target cells, we employed various cloning approaches including Gibson Assembly and constructed the pCas9gG-*Rosa26* vector (Figure 1A-a). This vector contains three independent expression modules, the *streptococcus pyogenes* Cas9 (spCas9) expression module, the double-nicking gRNA expression modules, and the eGFP expression module which allows for monitoring of transfection efficiency. To reduce off-target effects of single gRNA-guided Cas9 nuclease, a previously reported “paired nicking” strategy was employed, in which two sgRNAs targeted to adjacent sites on opposite DNA strands [44, 50], as two sgRNA, driven by U6 promoter, were designed to target the first intron of the *Rosa26* gene as reported (Figure 1A-a) [49].

For the donor template for CRISPR/Cas9-mediated homology-directed recombination, we constructed the targeting vector p*Rosa26*-TA (Figure 1A-b), containing 692bp upstream and 636bp downstream from the cleavage site of *Rosa26* locus. Between the two homology arms the vector contains the hEFH-driven SV40T-HygR bicistronic expression cassette linked by T2A self-cleaving peptide, which is flanked by FRT sites (Figure 1A-b). If the CRISPR/Cas9-mediated HDR is successful, the targeted cells should express SV40T and become resistant to hygromycin B (Figure 1A-c). Conversely, the SV40T-HygR cassette can be removed by FLP recombinase, leading to the expression of G418 resistance gene (see below).

To confirm if the above vectors could efficiently induce the integration of SV40T transgene into *Rosa26* locus in mouse cells, we transfected the targeting vector p*Rosa26*-TA and the CRISPR/Cas9-expressing vector p*Rosa26*-Cas9gG into mouse B16F10 cells. The GFP+ cells survived hygromycin B selection (Figure 1A-d). Genomic DNA PCR analysis of the stable cell pool was carried out to examine whether the *Rosa26*-targeting construct successfully integrated into the target site. Using the P1 primer that recognizes a genomic sequence outside of the downstream homology arm (or right arm) of the targeting vector, together with the G418-specific primer P2, we were able to detect the expected 791bp PCR product from the hygromycin B resistant B16F10 cells (Figure 1A-e), indicating the correct integration of the target sequence at the *Rosa26* locus. We further confirmed the 5´-end integrity of the targeting vector by detecting the presence of hEFH, SV40T and HygrR sequences with sequence-specific primers (Figure 1A-e). Lastly, we also used the retroviral-based immortalization vector SSR #41 as a control system in order to thoroughly compare the immortalization efficiency and FLP-mediated excision of SV40T cassette (Figure 1B). It is noteworthy that we also transfected the primary cells with single sgRNAs and found that single sgRNA seemingly exerted higher targeting efficiencies in these cells than double sgRNAs (data not shown) although thorough characterization of targeting specificity is needed.

### CRISPR/Cas9 HDR-mediated SV40T targeting system efficiently immortalizes primary mouse BMSCs

**Figure 2:**
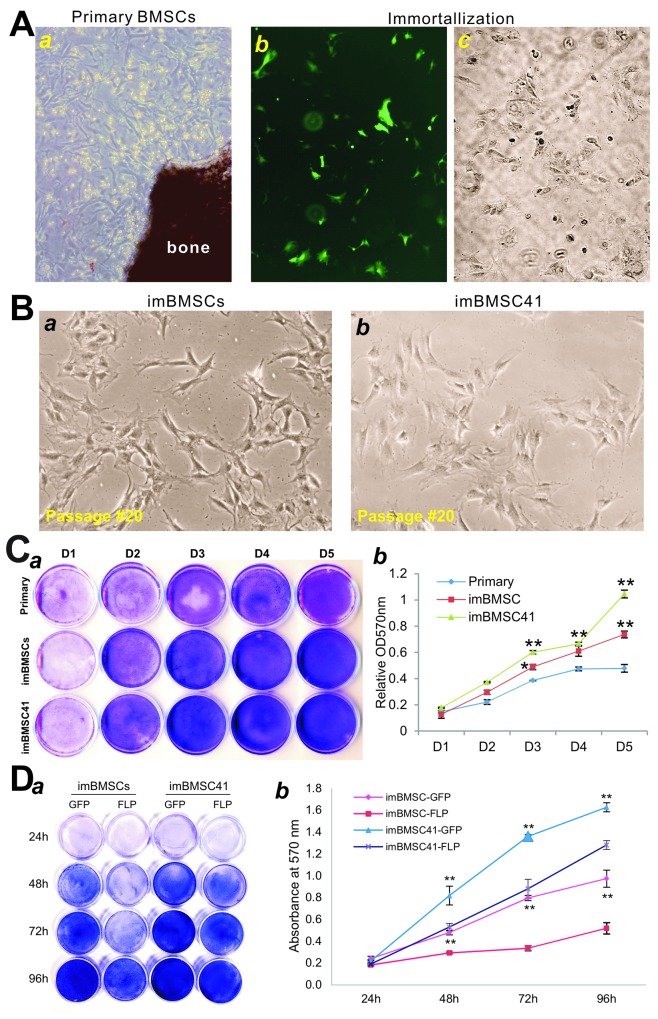
Immortalization of mouse bone marrow stromal stem cells (mBMSCs) **(A)** Primary mBMSCs were isolated from the femur bone morrow of 2-week old CD1 mice, and maintained in complete DMEM medium at day 3 ***(a)***. The primary mBMSCs (under passage 3) were co-transfected with pCas9gG-Rosa26 and pRosa26-TA plasmids. At 72 h post-transfection, a high level of GFP expression was detected in mBMSCs ***(b)***. The infected cells were selected in the presence of 0.1mg/ml hygromycin B. Surviving clones were observed at 5 days after selection ***(c)***. The resultant immortalized mBMSCs pool is designated as imBMSCs. The primary mBMSCs were also immortalized by using the SSR #41 retroviral vector as reported [47] and led to the establishment of imBMSC41 cell line. **(B)** The morphology of the reversibly immortalized mBMSC lines, imBMSCs ***(a)*** and imBMSC41 ***(b)***. Representative images of passage #20 are shown. **(C)** Cell viability and proliferation assay. The same numbers of primary mBMSCs, imBMSCs and imBMSC41 cells were seeded at low density and stained with Crystal Violet at the indicated time points ***(a)***. The stained cells were dissolved and quantitatively determined at A570nm ***(b)***. “^*^” p<0.05; “^**^”, p<0.01 compared with that of primary mBMSCs. Staining was done in triplicate and representative images are shown. **(D)** FLP-mediated removal of SV40 T antigen leads to decreased cell proliferation. Subconfluent imBMSCs and imBMSC41 cells were infected with Ad-FLP or Ad-GFP, and stained with Crystal Violet at the indicated time points ***(a)***, which were quantitatively determined at A570nm ***(b)***. Staining was done in triplicate and representative images are shown. “^**^”, p<0.01 compared with that of respective Ad-GFP-infected cells.

We next tested if the CRISPR/Cas9 HDR-mediated SV40T targeting system can effectively immortalize mBMSCs while retaining their multipotent properties. Primary mBMSCs were readily isolated from the femur of 2-week old CD1 mice (Figure 2A-a) and effectively co-transfected with pRosa26-TA and pCas9gG-Rosa26 plasmids (Figure 2A-b). When the transfected mBMSCs were selected in hygromycin B, stable cell clones were readily formed(Figure 2A-c). The pooled stable cells were continuously passaged and designated as imBMSCs (Figure 2B-a). Similarly, using the retroviral SSR #41 vector system as previously described [28, 36, 47], we established stable and immortalized mBMSC line, designated as imBMSC41 (Figure 2B-b).

We compared the proliferative activity of the two immortalized lines with that of primary mBMSCs, and found that while the primary mBMSCs significantly proliferated over the course of 5 days the immortalized lines (especially imBMSC41) grew at much higher rates. Crystal violet staining assay indicated that both imBMSCs and imBMSC41cells reached confluence at as early as day 3, while primary BMSCs reached confluence at day 5 even though all three lines started with a similar cell density (Figure 2C-a). Quantitative assessment of the stained cells confirmed the staining results that imBMSCs and imBMSC41 cells exhibited significantly higher proliferative activity at each time points than that of primary mBMSCs (Figure 2C-b). Interestingly, imBMSC41 cells had higher proliferation rate than that of imBMSCs at day 3 (p<0.05) and day 5 (p<0.001) (Figure 2C-b), which may be indicative of higher level of SV40T expression in imBMSC41 cells. Nonetheless, these results indicate that the primary mBMSCs were stably immortalized and maintain long-term proliferation capability.

We also tested if the immortalization phenotype could be effectively reversed by FLP recombinase. Using adenovirus-mediated effective expression of FLP, we found the Ad-FLP-infected imBMSCs grew significantly slower than that of Ad-GFP-infected counterpart at each time points (Figure 2D-a). While holding a similar trend but at a much lesser extent, Ad-FLP-infected imBMSC41 cells grew slower than that of Ad-GFP-infected imBMSC41 cells (Figure 2D-a). Quantitative analysis of the stained cells indicated that FLP-mediated removal of SV40T significantly decreased cell proliferation activities of the immortalized cells (p<0.001), while Ad-FLP-infected imBMSCs exhibited more significant decreases than that of Ad-FLP-infected imBMSC41 cells at 48h, 72h and 96h (Figure 2D-b). Consistent with the above observations, the imBMSC41 exhibited much higher proliferative activity than the imBMSCs (p<0.001). Moreover, the above results indicate that FLP-mediated removal of SV40T in the imBMSCs is more efficient than that of the imBMSC41 cells.

### The immortalization element in the imBMSCs can be removed by FLP recombinase more effectively than that in the imBMSC41 cells

**Figure 3:**
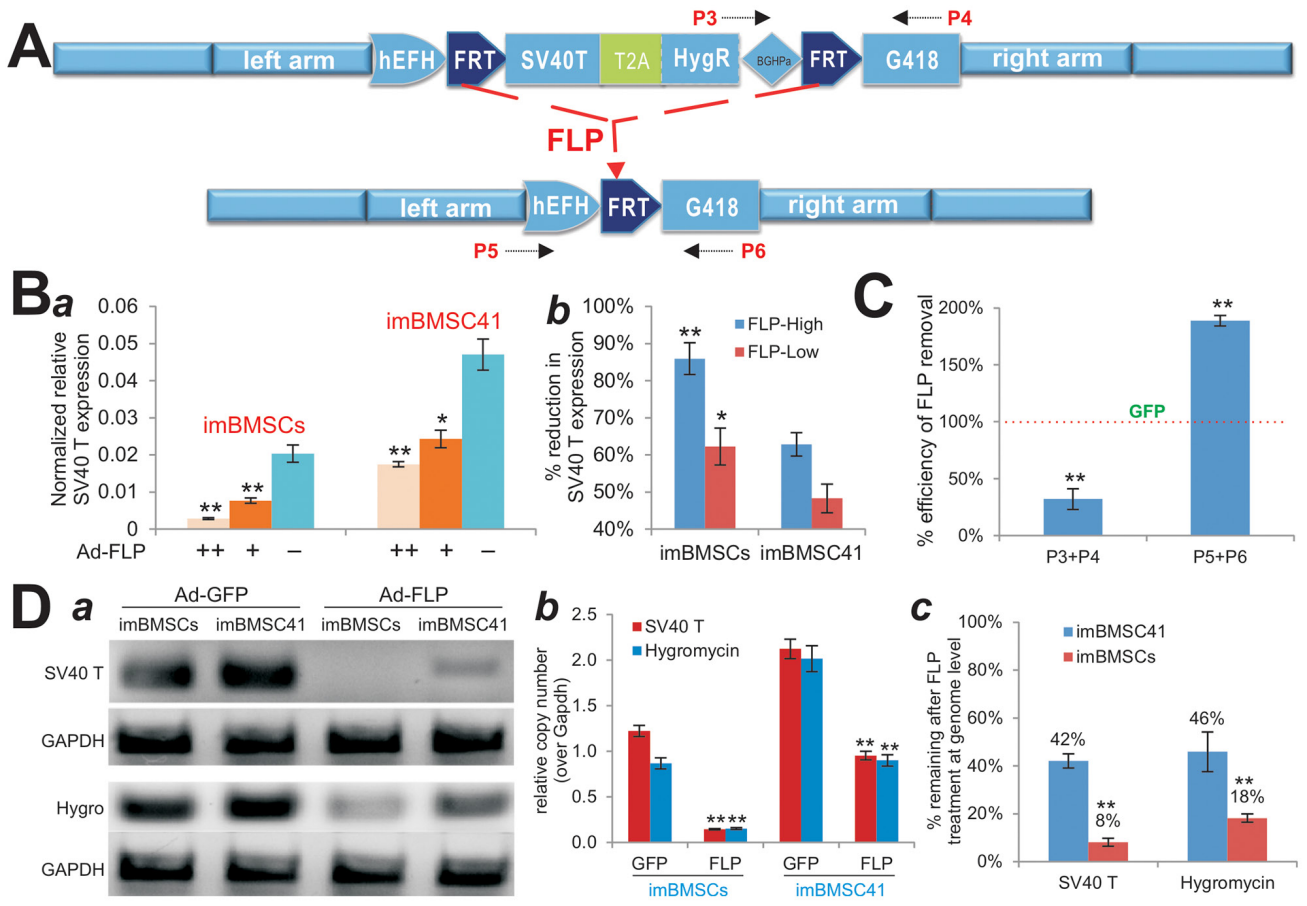
Effective removal of the transgenes integrated at the mouse *Rosa26* locus through CRISPR/Cas9-mediated HDR **(A)** Schematic representation of Flippase recombinase (FLP)-mediated excision of SV40T-HygR cassette from *Rosa26* locus. P3/P4 and P5/P6 are PCR primer pairs used to assess the efficiency of FLP-mediated excision. **(B)** FLP-mediated efficient removal of SV40 T antigen detected by TqPCR analysis. Subconfluent imBMSCs or imBMSC41 was infected with high or low titer of Ad-FLP or Ad-GFP control virus. At 5 days after infection, total RNA was isolated and subjected to TqPCR analysis of SV40 T antigen expression ***(a)***. *Gapdh* served as an internal control. The assays were done in triplicate. The % reduction of SV40 T antigen expression was also calculated and graphed ***(b)***. “^*^”, p<0.05; “^**^”, p<0.001. **(C)** & **(D)** Genomic PCR for the confirmation of FLP-mediated excision of SV40 T and Hygromycin cassette in the immortalized cells. The imBMSCs and imBMSC41 were infected with Ad-FLP or Ad-GFP for 5 days. Genomic DNA was isolated from the infected cells and subjected to TqPCR to assess the efficiency of FLP-mediated removal of SV40T and HygR sequences using the indicated primers. Percentage remaining after FLP treatment at genome level was calculated and graphed ***(c)***. *Gapdh* genomic primers were used as a normalization control or reference gene. All PCR reactions were done in triplicate. Representative sqPCR results are shown. “^**^”, p<0.001 compared with that of the Ad-GFP infected cells.

We next analyzed the distinct features of transgene expression and FLP-mediated reversibility between the imBMSCs and imBMSC41 cells. As expected, FLP recombinase should be able to remove the SV40T and HygR, which is marked by the disappearance of the P3/P4 fragment and the presence of P5/P6 fragment in genomic PCR analysis (Figure 3A). As reported above, Ad-FLP-infected imBMSCs grew significantly slower than that of Ad-GFP-infected counterpart at each time points (Figure 2D), indicating that immortalization phenotype of both lines are reversible to various extents by FLP recombinase.

We quantitatively analyzed the effect of FLP treatment on SV40T expression in both lines. When the imBMSCs and imBMSC41 cells were infected with low and high titers of Ad-FLP or Ad-GFP for 5 days, we found that the expression level of SV40T was significantly decreased in both lines in an Ad-FLP dose-dependent fashion (Figure 3B-a). It is noteworthy that the SV40T expression level in the imBMSC41 cells was approximately 2.3-fold of that in the imBMSCs (p<0.001), which may at least partially explain the higher proliferative activity of imBMSC41 cells. However, upon Ad-FLP infection SV40T expression in imBMSCs was shown to decrease by up to 86% (higher titer), compared with 63% reduction in imBMSC41 cells at the same Ad-FLP titer (Figure 3B-b). In fact, the FLP-mediated decrease in SV40T expression in imBMSCs was more profound than that in imBMSC41 cells both at high titer (p<0.001) and low titer (p<0.05) of Ad-FLP (Figure 3B-b). Quantitative PCR analysis of genomic DNA indicates that FLP-treated imBMSCs exhibited a significant decrease in P3/P4 PCR product, along with a significant increase in P5/P6 PCR product, compared with that of the GFP-treated imBMSCs (both p<0.001) (Figure 3C). These results further confirm that the CRISPR/Cas9 HDR-targeted immortalization element can be effectively removed by FLP recombinase in the imBMSCs.

We also compared the efficiency of the FLP-mediated removal of SV40T and HygR sequences at genome level in both cell lines. Semi-quantitative PCR analysis revealed that Ad-FLP infection effectively removed SV40T sequence in imBMSCs while residual amount of SV40T sequence was still detectable (Figure 3D-a). Similarly, Ad-FLP infection was shown to remove most of the HygR sequence in imBMSCs, but to a much lesser extent in imBMSC41 cells (Figure 3D-a). Quantitative PCR analysis indicated that FLP expression effectively removed both SV40T and HygR sequences in both cell lines, compared with that of GFP-transduced cells (p<0.001) (Figure 3D-b). By comparing with the genomic level of *Gapdh*, we found that the average copy numbers of SV40T/HygR genes were 1.045 in imBMSCs and 2.069 in imBMSC41 cells, respectively (Figure 3D-b), indicating that a single copy of the immortalization element was integrated into the Rosa26 locus through CRISPR/Cas9 HDR system in imBMSCs, while about two copies were randomly integrated in imBMSC41 cells. At least 92% of SV40T and 82% of HygR genes could be removed by FLP recombinase in imBMSCs, compared with 58% and 54% of these genes in imBMSC41 cells, respectively (Figure 3D-c), further confirming that the FLP-mediated removal of the immortalization element in imBMSCs is much more efficient than that in imBMSC41 cells (p<0.001).

### The immortalized BMSCs express most common MSC markers and retain the potential to differentiate into multiple lineages upon BMP9 stimulation

**Figure 4:**
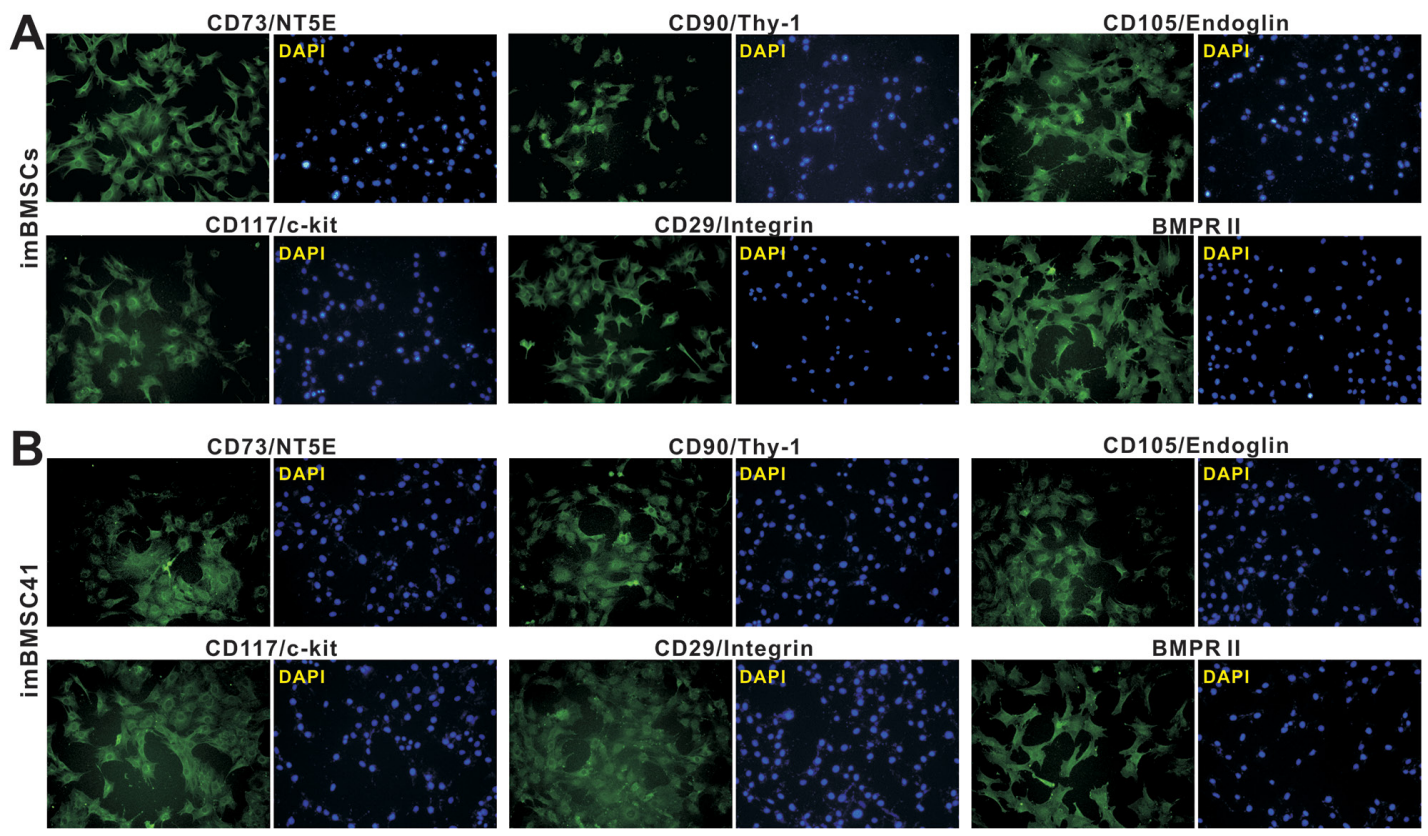
The imBMSCs and imBMSC41 cells are positive for most of the mesenchymal stem cell markers Subconfluent imBMSCs **(A)** and imBMSC41 **(B)** cells were stained for MSC markers as described in the Methods. Antibodies against CD73/NT5E, CD90/Thy-1, CD105/Endoglin, CD117/c-Kit, CD29/integrin β1, and BMPRII were from Santa Cruz Biotechnology. Minus primary antibodies and isotype IgG were used as negative controls (data not shown). Cell nuclei were counterstained with DAPI. Representative images are shown.

We next tested if the immortalized BMSC lines retain MSC features and functionalities. First, we examined the expression of common MSC markers in imBMSCs and imBMSC41 cells. While no single marker can be used to identify BMSC cells, several consensus MSC markers, such as CD73/NT5E, CD90/Thy-1, and CD105/Endoglin, were reported [28, 34, 35, 38, 39]. We found that the MSC markers were readily detected by immunofluorescence staining both in imBMSCs (Figure 4A top row) and imBMSC41 cells (Figure 4B top row). In addition, we found that both imBMSCs and imBMSC41 cells expressed other MSC and/or progenitor markers, including CD117/c-kit, CD29/Integrin β1 and BMPR II (Figure 4A,B, bottom row). Furthermore, the expression of hematopoietic stem cell markers CD45 and CD34 was not detected in both cell lines (data not shown). These results demonstrate that imBMSCs and imBMSC41 expressed most if not all of the common MSC markers, suggesting that these cells may retain MSC-like phenotypes.

**Figure 5:**
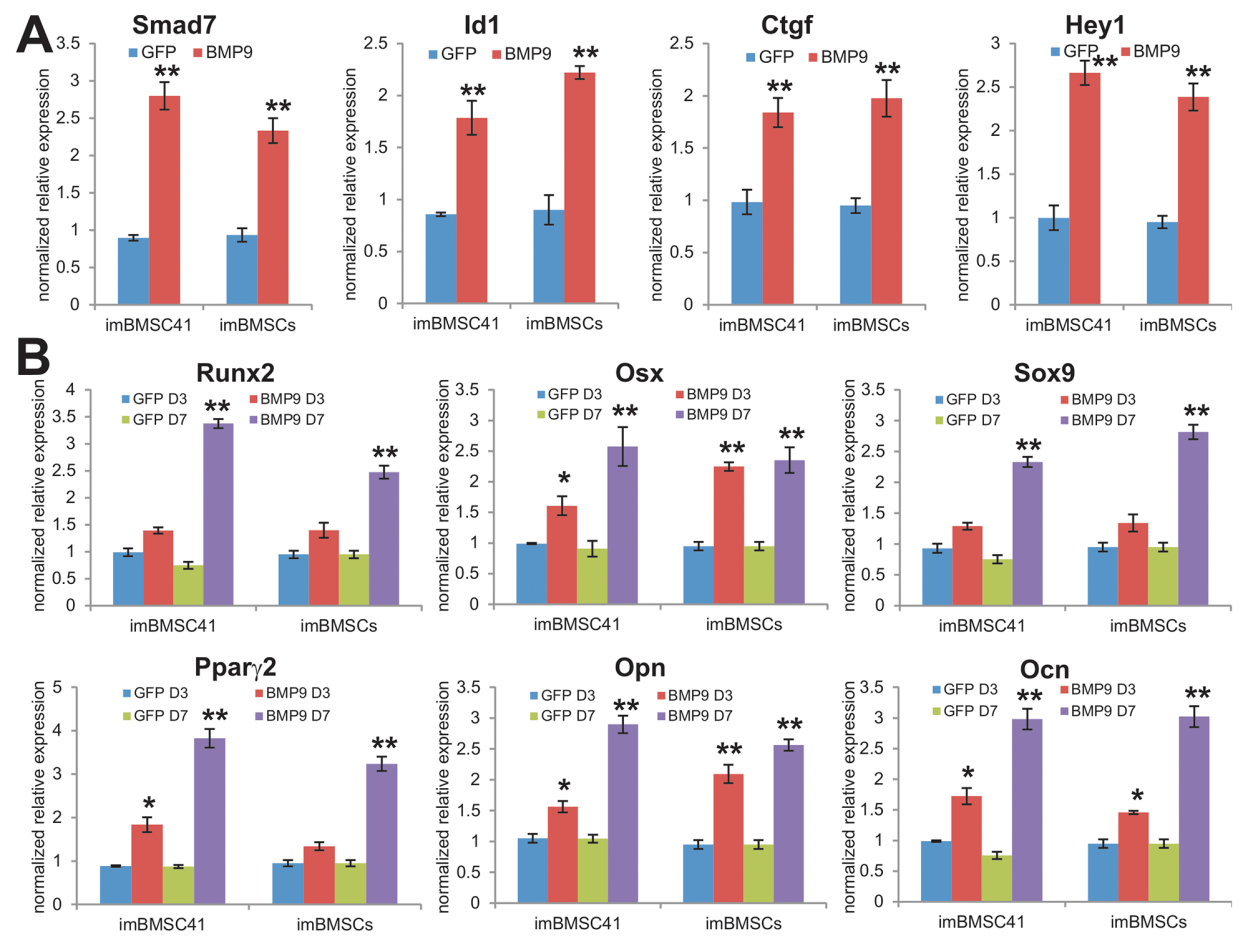
The immortalized BMSCs are responsive to BMP9-induced lineage-specific differentiation of MSCs **(A)** BMP9 induces the expression of early responsive genes in imBMSCs and imBMSC41 cells. Subconfluent imBMSCs and imBMSC41 cells were infected with Ad-BMP9 or Ad-GFP for 36h. Total RNA was isolated and subjected to TqPCR analysis using gene-specific primers for mouse *Smad7*, *Id1*, *Ctgf* and *Hey1*. **(B)** BMP9 induces the expression of multiple lineage regulators and osteogenic markers. Subconfluent imBMSCs and imBMSC41 cells were infected with Ad-BMP9 or AdGFP. Total RNA was isolated at the indicated time points and subjected to TqPCR analysis using gene-specific primers for mouse *Runx2*, *Osx*, *Sox9*, *Pparγ2*, *Opn* and *Ocn*. Expression of each target gene was calculated as a relative expression to *Gapdh* and represented as fold induction over control cells. Data are represented as mean ± SD of three independent experiments. “^*^” p< 0.05; “^**^”, p<0.001.

We previously demonstrated that BMP9 is a potent inducer of osteogenic, chondrogenic, and adipogenic differentiation of MSCs [22, 24, 51], and identified several important early responsive genes induced by BMP9 in MSCs such as *Smad7*, *Id1*, *Ctgf* and *Hey1* [52-55]. We infected imBMSCs and imBMSC41 cells with Ad-BMP9 or Ad-GFP for 36h, quantitative TqPCR analysis indicated BMP9 significantly up-regulated the expression of *Smad7*, *Id1*, *Ctgf* and *Hey1* (p<0.001) (Figure 5A). Furthermore, BMP9 was shown to effectively induce the expression of osteogenic regulators Runx2 and osterix (Osx), chondrogenic regulator Sox9 and adipogenic regulator Pparγ2 in both lines at day 3 and/or day 7 after BMP9 stimulation (Figure 5B). In addition, two osteogenic markers osteopontin (Opn) and osteocalcin (Ocn) were significantly induced by BMP9 in imBMSCs and imBMSC41 cells (Figure 5B). Collectively, these results demonstrate that the immortalized BMSCs retain MSC features.

### The immortalized BMSCs are highly responsive to BMP9-induced osteogenic signaling, which is not significantly affected by SV40 T antigen

**Figure 6:**
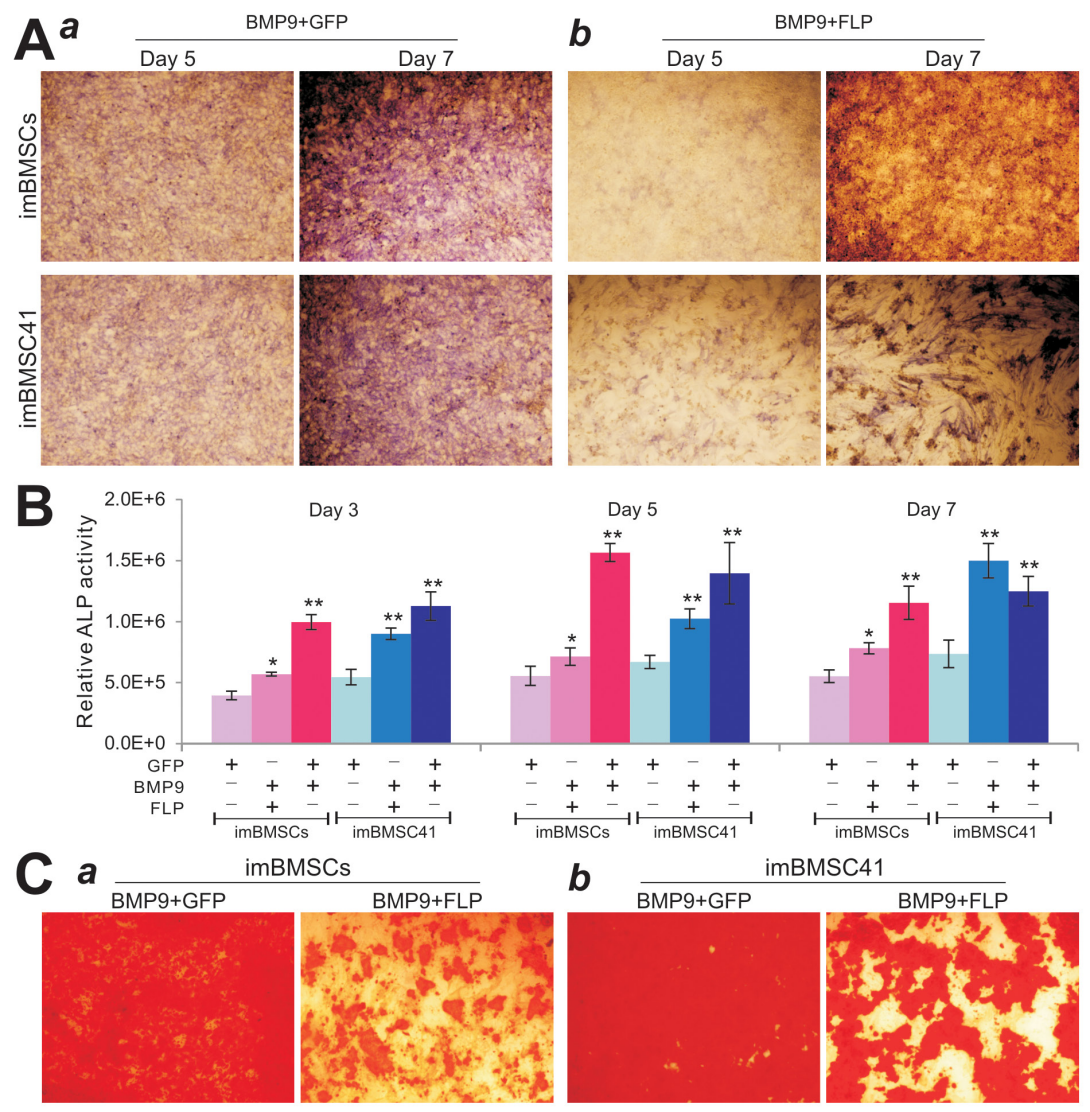
BMP9 induces effective osteogenic differentiation in the imBMSCs and imBMSC41 cells *in vitro* **(A)** & **(B)** BMP9 effectively upregulates early osteogenic marker alkaline phosphatase (ALP) activity in imBMSCs and imBMSC41 cells. Subconfluent imBMSCs and imBMSC41 cells were co-infected with AdBMP9 and Ad-GFP ***(a)*** or Ad-FLP ***(b)***. ALP activity was determined histochemically (A) or quantitatively (B) at the indicated time points. All assays were done in triplicate and representative images are shown. “^*^” p<0.05; “^**^”, p<0.001 compared with that of Ad-GFP groups. **(C)** The effect of FLP-mediated removal of SV40T on later osteogenic differentiation of the imBMSCs and imBMSC41 cells. Subconfluent imBMSCs ***(a)*** and imBMSC41 ***(b)*** were co-infected with Ad-BMP9 and Ad-FLP or Ad-GFP and maintained in mineralization medium for 10 days. Cells were fixed and stained with Alizarin Red S. The staining was done in triplicate and representative images are shown.

We previously demonstrated that BMP9 is one of the most potent osteogenic factors [19, 21, 22]. Here, we analyzed if the immortalized BMSCs were responsive to BMP9 stimulation and/or the differentiation potential would be affected by FLP-mediated removal of SV40T. When imBMSCs and imBMSC41 cells were co-infected with Ad-BMP9 and Ad-FLP or Ad-GFP, we found the BMP9-induced alkaline phosphatase (ALP) activity significantly decreased in Ad-FLP transduced imBMSCs and imBMSC41 cells, compared to that of Ad-GFP-infected cells (Figure 6A-a,b). Quantitative ALP analysis also confirmed that the FLP-mediated removal of SV40T led a significant decrease in ALP activity in both imBMSCs and imBMSC41 cells at multiple time points (Figure 6B). We further determined the effect of FLP-mediated removal of SV40T on late stage osteogenic differentiation and found that FLP-mediated removal of SV40 T antigen reduced the BMP9-induced matrix mineralization in both lines, as assessed by Alizarin Red S staining, compared to that of the AdGFP-transduced cells (Figure 6C-a,b). While it remains to be further investigated why FLP-mediated removal of SV40T in both lines diminishes BMP9-induced osteogenic activity *in vitro*, one possible explanation is that the removal of SV40T in these cells may decrease their proliferative activity and hence significantly reduce the progenitor pools for osteogenic differentiation. Nonetheless, these results demonstrate that the immortalized BMSCs were responsive to BMP9-induced osteogenic signaling and that the expression of SV40 T antigen does not prevent the immortalized BMSCs from undergoing osteogenic lineage-specific differentiation.

**Figure 7:**
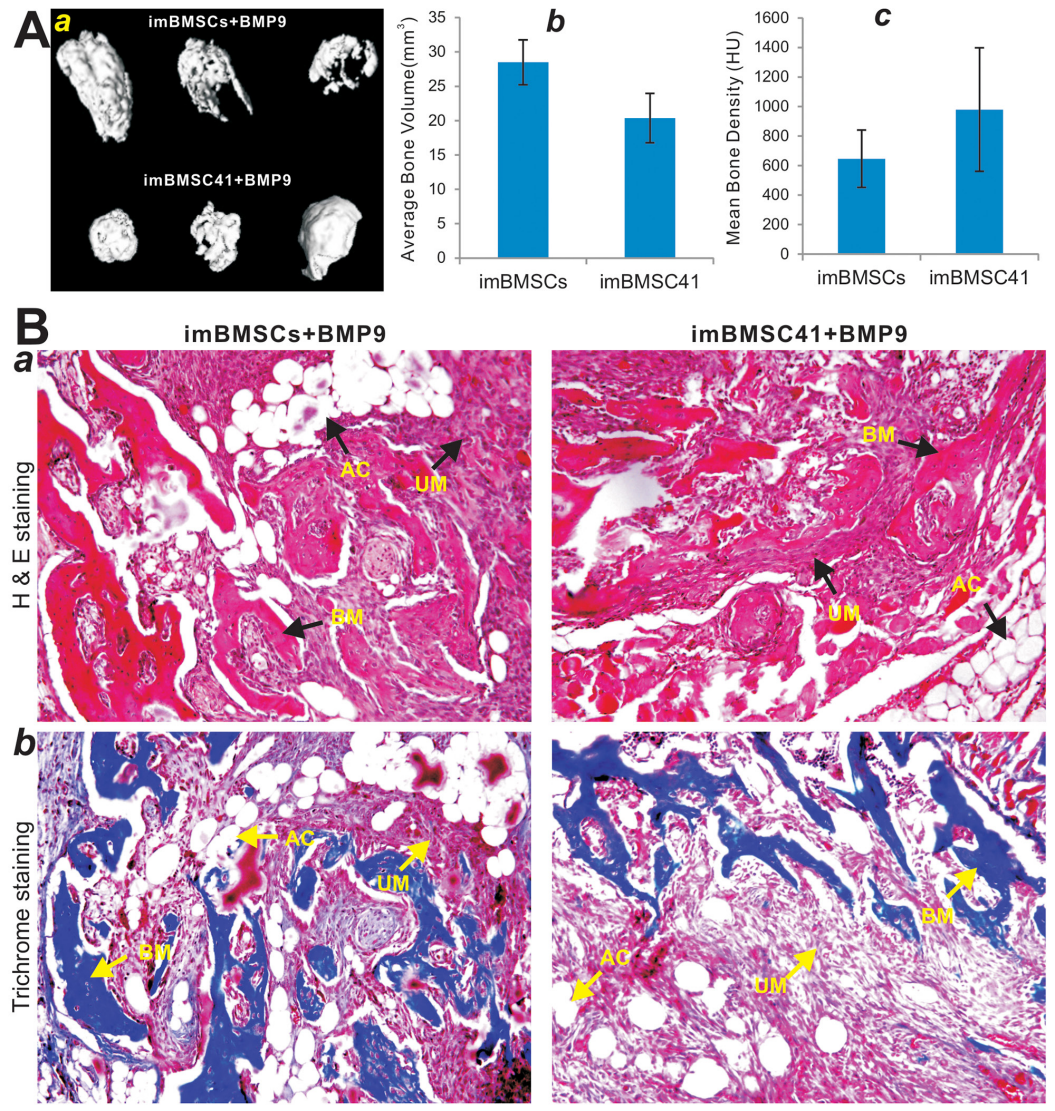
BMP9 induces robust ectopic bone formation from the imBMSCs and imBMSC41 cells *in vivo* **(A)** Subconfluent imBMSCs and imBMSC41 were infected with Ad-BMP9 or Ad-GFP for 36h. The infected cells were collected and injected into the flanks of athymic mice subcutaneously (n=5 per group). At 4 weeks after implantation, the subcutaneous bony masses at the injection sites were retrieved and fixed for μCT imaging. Representative 3D iso-surface reconstruction images for bone masses retrieved from subcutaneous injection with Ad-BMP9 infected cells ***(a)***. No masses were formed at the sites injected with the cells infected with Ad-GFP. The average bone volume ***(b)*** and mean bone mineral density ***(c)*** of the ectopic bone masses were analyzed using the software Amira 5.3. **(B)** H&E and Trichrome staining. The masses retrieved from subcutaneous injection with Ad-BMP9 infected imBMSCs and imBMSCs cells were fixed, decalcified, and subjected to H & E ***(a)*** and Trichrome ***(b)*** staining. Representative images are shown. For the Trichrome staining, decalcified bone matrix stained dark red, cartilage matrix stained blue. BM, bone matrix; AC, adipocyte; UM, undifferentiated MSCs.

Lastly, we conducted *in vivo* experiments to determine whether the immortalized BMSCs were tumorigenic and could fully differentiate into bone cells upon BMP9 stimulation. Using our previously established stem cell implantation assay [28, 51], we infected subconfluent imBMSCs and imBMSC41 cells with Ad-BMP9 or Ad-GFP, and injected subcutaneously into the flanks of athymic nude mice for 4 weeks. No recoverable masses were detected in both cell lines infected with Ad-GFP, suggesting that the immortalized BMSCs may be not tumorigenic or exhibit limited tumorigenic potential. However, robust bony masses were retrieved from Ad-BMP9 transduced imBMSCs and imBMSC41 cells (Figure 7A-a) although no significant differences in average bone volume and mean bone density were found between the two cell lines as determined by μCT imaging analysis (p>0.05) (Figure 7A-b,c). Accordingly, histological analysis of the retrieved masses revealed that BMP9-transduced imBMSCs and imBMSC41 cells formed robust osteoid matrix and trabecular bone, while adipocytes and to a much lesser extent, chondrocytes, were also observed within the retrieved bony masses (Figure 7B-a). It is noteworthy that significant amounts of undifferentiated BMSCs were also observed (Figure 7B-a), indicating that longer time points may be needed to allow the remaining progenitors to become fully differentiated. Trichrome staining further confirmed that BMP9 was able to induce the formation of mature bone and well-mineralized osteoid matrix (Figure 7B-b). It is noteworthy that we compared the *in vitro* and *in vivo* osteogenic activities among early passage primary BMSCs and the immortalized BMSCs lines and did not find any significant differences in their response to BMP9 stimulation (data not shown), suggesting that SV40 Tag-mediated immortalization may not significantly affect the lineage-specific differentiation potential of BMSCs. Taken together, these *in vitro* and *in vivo* results strongly suggest that both imBMSCs and imBMSC41 cells may give rise to osteogenic, chondrogenic and adipogenic lineages.

## DISCUSSION

In order to accomplish the establishment of reversibly and/or conditionally immortalized BMSCs for basic and translational research, we have taken advantage of the CRISPR/Cas9-based site-specific HDR and targeted the immortalizing gene SV40T to the safe harboring site at the *Rosa26* locus of mouse genome. We demonstrated that CRISPR/Cas9 HDR-mediated SV40T targeting system can efficiently immortalize primary mouse BMSCs and developed the reversibly immortalized BMSCs or imBMSCs. For a comparison, we also immortalized BMSCs with the retroviral vector SSR #41 and established the imBMSC41. Compared with primary mBMSCs, both imBMSCs and imBMSC41 exhibited long-term proliferative capability although imBMSC41 cells had significantly higher proliferation rate. Quantitative analysis indicated that imBMSC41 cells expressed approximately 130% higher level of SV40T than that of imBMSCs. High expression level of FLP recombinase led to approximately 86% reduction of SV40T expression in imBMSCs, compared with 63% in imBMSC41 cells. Quantitative genome PCR indicated that the average genomic copy number of SV40T and hygromycin was approximately 1.05 for imBMSCs and 2.07 for imBMSC41 cells, whereas genomic DNA analysis further demonstrated that FLP transduction efficiently removed the immortalization cassette from the *Rosa26* locus in mBMSCs. Furthermore, FLP expression in imBMSCs was shown to remove approximately 92% of SV40T at genome DNA level, compared with 58% of that in imBMSC41 cells under the same condition. These results strongly indicate that CRISPR/Cas9-mediated immortalization of BMSCs is target site-specific and can be more effectively reversed than that of retrovirus-mediated random integration. Nonetheless, both imBMSCs and imBMSC41 cells expressed MSC markers and were highly responsive to BMP9-induced osteogenic, chondrogenic and adipogenic differentiation *in vitro* and *in vivo*. Furthermore, we demonstrated that the presence of SV40T did not prevent BMP9-induced osteogenic differentiation and that the immortalized BMSCs were non-tumorigenic *in vivo*.

Our results reinforce the important utility of CRISPR/Cas9-mediated site-specific genome-editing technology in biomedical research. We previously used retroviral vector and *piggyBac* transposon-mediated expression of SV40T to immortalize multiple progenitor cells [28-39]. While effective and successful, retroviral or transposon-mediated random integration of immortalizing genes may possess detrimental effects in host genome, and is significantly less effective to be removed by Cre or FLP recombinase. While mouse *Rosa26* locus has been used as a “safe harbor” locus for targeted integration because this site is not susceptible to gene silencing effects and provides improved targeting efficiency and ubiquitous transgene expression without alteration of the cell viability or phenotype [45, 46], it is conceivable that more “safe harbor” sites can be used as CRISPR/Cas9 target sites.

Several strategies have been developed to immortalize primary cells, including somatic fusion, stable expression of viral oncogenes, hTERT and/or inactivation of tumor suppressor genes [27, 56-59]. As one of the most commonly-used immortalizing genes, SV40T exerts immortalizing properties without fully transforming the cells [60]. We previously used SV40T to immortalize various sources of progenitor cells without detecting any tumorigenic effect [28-39]. Other commonly used oncogenes include c-Myc, KRas, CDK4, Bmi-1, cyclin D1, HPV 16 E6/E7, and Telomerase (TERT) while most of them require more than one oncogene to work together to immortalize primary cells [59]. Based on our experience, SV40T is sufficient and effective to immortalize primary mouse cells, although a combination of SV40T and hTERT may be more efficient in maintaining long-term proliferation of primary human cells, especially for human progenitor cells.

Immortalized bone marrow stromal cells can be used as a promising alternative cell source of primary MSCs for basic and translational studies. One such line of investigation is to study bone morphogenetic protein (BMP)-induced osteogenic signaling and the potential use of BMPs in regenerative medicine. BMPs are considered the most potent osteoinductive factors [19, 20]. Through a comprehensive analysis of the 14 types of BMPs’ osteogenic activities, we found that BMP9 (also known as growth differentiation factor 2, or GDF2) is among the most osteogenic BMPs that induce osteoblastic differentiation of MSCs [19, 21-24]. We demonstrated that BMP9 is resistant to naturally occurring antagonist noggin, which may at least partially contribute to its potent osteogenic activity [61]. We further demonstrated that TGFβ/BMP type I receptors ALK1 and ALK2 are essential for BMP9-induced osteogenic signaling in MSCs [62]. Mechanistically, BMP9 has been shown to induce osteogenic differentiation of MSCs by regulating a panel of important downstream targets [52-55, 63], as well as through cross-talk with other important signaling pathways [64-69]. Thus, using BMP9-expressing progenitor cells, such as BMSCs, may promote bone regeneration in large bony defects and/or fracture nonunion in clinical settings [23, 24, 70].

In summary, we established the reversibly immortalized imBMSCs by exploiting CRISPR/Cas9 HDR mechanism. The mBMSCs were successfully immortalized by targeting SV40T into the *Rosa26* locus. The resultant imBMSCs retained MSC features both *in vitro* and *in vivo*. Furthermore, the CRISPR/Cas9 HDR-immortalized BMSCs can be reversed more effectively by the FLP recombinase, compared to BMSCs immortalized with retroviral vector-based random integrations. Moreover, we demonstrated that the presence of SV40T did not prevent BMP9-induced osteogenic differentiation and that the immortalized BMSCs were non-tumorigenic *in vivo*. Collectively, the engineered imBMSCs should be used as a valuable resource for basic and translational research in the fields of MSC biology and regenerative medicine.

## MATERIALS AND METHODS

### Cells culture, enzymes and chemicals

HEK-293 and B16F10 were obtained from ATCC (Manassas, VA). The 293pTP and RAPA cells were derived from HEK-293 cells as described [71, 72]. All cells were maintained at 37°C with 5% CO^2^ in Dulbecco’s modified eagle medium (DMEM) supplemented with 10% (v/v) fetal bovine serum (FBS, Gemini Bio Products, West Sacramento, CA), 2mM L-glutamine, 100 U/ml penicillin and 100 mg/ml streptomycin [73, 74]. All restriction enzymes used in cloning, the Phusion High-Fidelity PCR kit and the Gibson Assembly Master Mix were from New England Biolabs (Ipswich, MA, USA). Oligonucleotides were synthesized by IDT (Coralville, IA). Unless indicated otherwise, all chemicals were purchased from Sigma-Aldrich (St. Louis, MO) or Thermo Fisher Scientific (Waltham, MA).

### Construction of the pCas9gG-Rosa26 vector that expresses spCas9 and double-nicking sgRNAs targeting *Rosa26* locus

As illustrated in Figure 1A-a, the pCas9gG-Rosa26 vector was constructed on the base of our homemade vector pU6CGR, which contains U6 promoter to drive small guide RNA (sgRNAs) expression. First, the CAG-spCas9 expression cassette was PCR amplified from pX330 (Addgene plasmid #42230), resulting in pCas9(CAG-spCas9-U6-EF1-eGFP-PA). Gibson Assembly reaction [75] was carried out to knockout the BmsI site in spCas9 cDNA sequence. To express dual sgRNAs that target the *Rosa26* locus, we subcloned the fragments containing U6 promoter and sgRNA scaffold into the above vector. Two previously characterized Rosa26-targeting sgRNAs [49] were chosen and subcloned into the BsmI and BbsI sites, respectively: sgRNA1, GCG CAC TAG ACG TTG AGG TCagg and sgRNA2, GAA GAT GGG CGG GAG TCT TCtgg, where the PAM sequences are in lower case. Lastly, a CMV-driven eGFP expression cassette was subcloned into the vector for monitoring transfection efficiency. The final construct was designated as pCas9gG-Rosa26, which is used to deliver constitutive expression of spCas9 and a pair of gRNAs to produce dual gRNA-guided double-nicking in mouse *Rosa26* locus. All cloning junctions and critical sequences were sequencing verified. Details about the vector construction are available upon request.

### Construction of the donor vector pRosa26-TA that contains the *Rosa26* homologous recombination arms and expresses SV40 T antigen (SV40T) and hygromycin resistance gene (HygR) flanked with FRT sites

The donor vector pRosa26-TA was constructed on the base of our homemade vector pMOK. Briefly, the 600-700bp fragments of *Rosa26* left and right homologous arms were PCR amplified from mouse genomic DNA and subcloned into the EcoRI/AfeI and MluI/BspDI sites of pMOK, respectively. Gibson Assembly reactions were carried out to assemble the hEFH-driven SV40T-T2A-HygR expression cassette (Figure 1A-b). The overlapping SV40T and HygR fragments with 50bp overlapping sequences on each side, along with T2A peptide sequence preceding the HygR coding region, were PCR amplified from pMPH86 [39]. Furthermore, the SV40T-T2A-HygR cassette was flanked with FRT sites, followed by the coding region of G418 resistance gene for negative selection after FLP treatment. All cloning junctions, PCR amplified sequences, and other critical sequences were sequencing verified. Details about the vector construction are available upon request.

### Isolation and culture of primary mouse bone marrow stromal stem cells (mBMSCs)

All animal studies were conducted by following the NIH guidelines approved by Institutional Animal Care and Use Committee (IACUC). Isolation and culture of primary mBMSCs from murine bone marrow were performed as previously described [76]. Briefly, 2-week old male CD1 mice (obtained from The University of Chicago Transgenic Core Facility) were euthanized. The connective tissues were removed from the femur and rinsed with sterile PBS. Both ends of the femur were cut, and the marrow plug was flushed out with syringe containing complete DMEM medium. The marrow plugs were washed in complete DMEM and plated into 100mm cell culture dishes at 37°C. After 3h incubation, the non-adherent cells were removed by replacing the medium with fresh complete medium. The medium was changed every 8h for the first 72h of culture. The cultured primary mBMSC cells were maintained in complete DMEM and used for immortalization experiments.

### Establishment of reversibly immortalized mouse BMSCs (imBMSCs) using the CRISPR/Cas9-mediated SV40T expression in *Rosa26* locus

Early passage BMSCs (<3 passages) were seeded in 25 cm^2^ flasks and co-transfected with 6 μg of pRosa26-TA and 2 μg of pCas9gG-Rosa26 using 75 μg PEI (Polysciences Inc, Warrington, PA) as described [77, 78]. At 36h after transfection, cells were subjected to hygromycin B selection (at 0.1 mg/mL) for 7 days. A stable cell pool was obtained and continuously passaged for further analysis. The resultant stable line was designated as imBMSCs.

In order to compare the biological features and FLP-mediated reversibility, we also immortalized primary mBMSCs with a retroviral immortalization system described previously [28-32, 35, 36, 47]. Specifically, we infected early passage primary mBMSCs with packaged SSR #41 retroviral vector, which expresses SV40 T antigen flanked with FRT sites [47]. Experimentally, early passage mBMSCs (<3 passages) were seeded in 25 cm^2^ flasks and infected by SSR #41 retrovirus with 3 rounds (e.g., 3-4h intervals between rounds). Stable cell pool was obtained by selecting the infected cells with hygromycin B (0.1 mg/mL) for 7 days, and was continuously passaged for further analysis. The resultant stable pool was designated as imBMSC41. Aliquots of the varied passages of imBMSCs or imBMSC41 were kept in liquid nitrogen tanks.

### Generation and amplification of recombinant adenoviruses expressing BMP9, FLP recombinase and GFP

Recombinant adenoviruses were generated using the AdEasy technology as described [79, 80]. Briefly, the coding regions of human BMP9 and flippase recombinase (FLP) were PCR amplified, subcloned into an adenoviral shuttle vector, and subsequently used to generate recombinant adenoviruses in HEK-293, 293pTP or RAPA cells as described [71, 72]. The resulting adenoviruses were designated as Ad-BMP9 and Ad-FLP, both of which also express GFP as the marker for monitoring infection efficiency. An analogous adenovirus expressing only GFP (Ad-GFP) was used as mock virus control. For all adenovirus infections, polybrene (8 μg/mL) was added to the culture medium in order to enhance adenoviral transduction efficiency [81].

### Total RNA isolation and touchdown quantitative real-time PCR (TqPCR)

Total RNA was isolated using TRIZOL Reagents (Invitrogen) and reverse transcribed using hexamer and M-MuLV reverse transcriptase (New England Biolabs, Ipswich, MA). The resulting cDNA products were diluted 10- to 100-fold and used as PCR templates. TqPCR was carried out by using SYBR Green-based TqPCR analysis on a CFX-Connect unit (Bio-Rad Laboratories, Hercules, CA). PCR primers were designed by using Primer3 program and were listed in Supplementary Table 1. Quantitative real-time PCR analysis was carried out by using our recently optimized TqPCR protocol [82]. Briefly, the PCR reactions were carried out by using a touchdown protocol: 95°C×3min for one cycle; 95°C×20 sec, 66°C×10 sec for 4 cycles, with 3°C decrease per cycle; followed by 95°C×10 sec, 55°C×15 sec, 70°C×1 sec for 40 cycles, followed by plate read. All reactions were done in triplicate. The TqPCR amplification was confirmed by performing the melting curve test and observing a single peak for each gene. *Gapdh* was used as a reference gene.

### Genomic DNA characterization at the *Rosa26* targeting sites

Genomic DNA was extracted from imBMSCs and imBMSC41 cells by using alkaline lysis method. The primers P1 and P2 were designed from the mouse genomic sequence immediately outside of the right homologous arm and the G418 doing sequence in the target vector (Figure 1A-c). The presence or absence of hEFH, SV40 T and hygromycin sequences was also assessed by PCR using sequence-specific primers (Supplementary Table 1). The PCR reactions were carried out by using a touchdown protocol: 95°C×3min for one cycle; 95°C×20 sec, 68°C×30 sec, 72°C×40 sec for 13 cycles, with 1°C decrease per cycle; followed by 95°C×20 sec, 55°C×30 sec, 72°C×40 sec for 20–25 cycles, depending on the transcript abundance. PCR products were confirmed on 1.2 % agarose gels.

The relative copy numbers of the transgenes were determined by TqPCR analysis. The qPCR primers were designed to detect SV40T and Hygromycin genes. TqPCR analysis was carried out as described [82]. Primers for the *Gapdh* promoter were used as an internal control.

### Immunofluorescence staining

Immunofluorescence staining was performed as described [33, 83, 84]. Briefly, exponentially growing cells were fixed with 4% paraformaldehyde (PFA) for 10 min, washed with PBS and permeabilized with 1% NP-40 for 10min at room temperature. After being blocked with 10% donkey serum (Jackson Immuno-Research Laboratories, West Grove, PA) for 1h at room temperature, cells were incubated with various primary antibodies, including CD29, CD73, BMPRII, CD90, CD117/c-kit, CD105/endoglin, or BMPR-II antibody (all from Santa Cruz Biotechnology) for 1h at room temperature. Cells were washed with PBS and incubated with FITC-labeled secondary antibodies (Jackson ImmunoResearch Laboratories) for 30 min. DAPI (Invitrogen) was used to visualize nuclei. Stains were examined under a fluorescence microscope. Negative control cells were performed under the same conditions without primary antibodies. Representative images from at least three independent staining experiments are shown.

### Early osteogenic marker alkaline phosphatase (ALP) activity assay

ALP activity was assessed quantitatively with the modified Great Escape SEAP Chemiluminescence assay kit (BD Clontech) and qualitatively with histochemical staining assay (using a mixture of 0.1 mg/ml napthol AS-MX phosphate and 0.6 mg/ml Fast Blue BB salt) as previously described [65, 68]. Each assay condition was performed in triplicate, the results were repeated at least three independent experiments and was normalized by total cellular protein concentrations among the samples.

### Matrix mineralization assay (Alizarin Red staining)

The formation of mineralized matrix nodules was stained by using Alizarin Red S staining as described previously [67, 85]. Briefly, cells were co-infected with AdBMP9 and Ad-GFP or Ad-FLP, and cultured in the presence of ascorbic acid (50 μg/mL) and β-glycerophosphate (10 mM). At 10 days after infection, cells were fixed with 0.05% (v/v) glutaraldehyde at room temperature for 10 min and washed with distilled water. The fixed cells were incubated with 0.4% Alizarin Red S for 5 min, followed by extensive washing with distilled water. The staining of calcium mineral deposits was recorded under a bright field microscope. Each assay condition was done in triplicate.

### Subcutaneous implantation of MSCs for ectopic bone formation

The use and care of experimental animal studies was approved by the Institutional Animal Care and Use Committee (IACUC). Stem cell-based ectopic bone formation was performed as previously described [63, 86]. Briefly, imBMSCs and imBMSC41 were infected with Ad-BMP9 or Ad-GFP for 36h, collected and resuspended in 80μL of phosphate-buffered saline (PBS) for subcutaneous injection (5×10^6^ cells/site) into the flanks of athymic nude (nu/nu) mice (5 animals per group, 4-6 weeks old, male; Harlan Laboratories, Indianapolis, IN). At 4 weeks after implantation, animals were sacrificed, and the implantation sites were retrieved for microcomputed tomography (μCT) imaging, histologic evaluation and other special staining (see below).

### Micro-CT (μCT) analysis

All retrieved specimens were fixed in 10% (v/v) formalin and imaged using the μCT component of the GE triumph (GE Healthcare) trimodality preclinical imaging system. The imaging data were analyzed with Amira 5.3 software (Visage Imaging, Inc.). The 3D volumetric values and mean bone density were determined as described [87].

### Histological evaluation (H&E) and trichrome staining

Retrieved tissues were fixed in 10% (v/v) formalin overnight, then decalcified and paraffin embedded. Serial sections of the embedded specimens were mounted onto slides, deparaffinized and rehydrated in a graduated fashion. H & E and Masson’s Trichrome stains were carried out as previously described [88].

### Statistical analysis

All quantitative assays were performed in triplicate and/or in three independent batches. Statistical analysis was carried out using Microsoft Excel program. Data were expressed as mean ± SD. Statistical significances were determined by one-way analysis of variance and the student’s t test. A value of p<0.05 was considered statistically significant.

## SUPPLEMENTARY MATERIALS TABLE




