# Supplementary material for: CRISPR/Cas9-mediated reversibly immortalized mouse bone marrow stromal stem cells (BMSCs) retain multipotent features of mesenchymal stem cells (MSCs)
Source: Oncotarget. 2017 Dec 5;8(67):111847–65. doi: 10.18632/oncotarget.22915 (PMC5762364; doi:10.18632/oncotarget.22915)
Supplement: Supplementary file 1 [file oncotarget-08-111847-s001.pdf]

CRISPR/Cas9-mediated reversibly immortalized mouse bone marrow stromal stem cells (BMSCs) retain multipotent features of mesenchymal stem cells (MSCs)

SUPPLEMENTARY MATERIALS

Supplementary Table 1: List of PCR and Cloning Primers

See Supplementary File 1
